# Editorial: Advances in Alzheimer’s disease diagnostics, brain delivery systems, and therapeutics

**DOI:** 10.3389/fmolb.2023.1162879

**Published:** 2023-03-17

**Authors:** Ashok Iyaswamy, Karthick Vasudevan, Selvaraj Jayaraman, Ravindran Jaganathan, Abhimanyu Thakur, Raymond Chuen-Chung Chang, Chuanbin Yang

**Affiliations:** ^1^ School of Chinese Medicine, Mr. And Mrs. Ko Chi Ming Centre for Parkinson’s Disease Research, Hong Kong Baptist University, Kowloon, Hong Kong SAR, China; ^2^ Department of Biochemistry, Karpagam Academy of Higher Education, Coimbatore, India; ^3^ Department of Biotechnology, REVA University, Bangalore, India; ^4^ Centre of Molecular Medicine, Department of Biochemistry, Saveetha Dental College and Hospitals, Chennai, Tamil Nadu, India; ^5^ Preclinical Department, Faculty of Medicine, Royal College of Medicine Perak, Universiti Kuala Lumpur, Perak, Malaysia; ^6^ Pritzker School of Molecular Engineering, Ben May Department for Cancer Research, The University of Chicago, Chicago, IL, United States; ^7^ Laboratory of Neurodegenerative Diseases, School of Biomedical Science, LKS Faculty of Medicine, The University of Hong Kong, Pokfulam, Hong Kong SAR, China; ^8^ Department of Geriatrics, Shenzhen People’s Hospital, The Second Clinical Medical College, Jinan University, The First Affiliated Hospital, Southern University of Science and Technology, Shenzhen, China

**Keywords:** neurodegenerative diseases, Alzheiemer’s disease, diagnostics, brain delivery systems, therapeutics

Neurodegenerative diseases (ND) have caused a wide range of distress and social problems in the aging population and affect millions of people worldwide (Hansson, 2021). Among the wide variety of NDs, Alzheimer’s disease (AD) and Parkinson’s disease (PD) are the most widely spread brain diseases causing progressive neuronal damage, memory loss, and movement and coordination disorders in the aging population (Sun and Roy, 2021). In recent years, the advancement of preclinical research has unveiled a number of biomarkers for the diagnosis of NDs and several therapeutic strategies have been researched in clinical trials into AD and PD (Cai and Jeong, 2020; Hansson, 2021). These innovations have led us to discover novel methods for the accurate diagnoses of NDs and the precise therapeutics for effective care (Sun and Roy, 2021). Recently, many gene-based therapies, pharmacological approaches, and extracellular vesicle (EV) based brain delivery systems have created new avenues for the diagnosis and treatment of AD and PD (Izadpanah et al., 2018). With the aim of bringing together innovative and novel research articles for our Research Topic, we invited an enormous number of scientists and academicians around the world to submit their research articles to this Research Topic entitled “Advances in Alzheimer’s Disease Diagnostics, Brain Delivery Systems, and Therapeutics”. We welcomed different research submissions pertaining to the application of advanced technology, inter-disciplinary approaches, and the strategies that have helped the understanding of the prognosis and pathogenesis of NDs. Among the submitted articles, four manuscripts were accepted which matched the Research Topic and are published in our Research Topic. In this editorial, we will summarise the published articles and their findings with concluding remarks for the future scope of research advancements in the diagnostics and therapeutics of NDs.

Over the years, Traditional Chinese Medicine (TCM) has been in practiced in East Asia for the treatment of neurological disorders like AD and PD using herbal formulations (Iyaswamy et al., 2021) and phytochemicals (Durairajan et al., 2017; Wang et al., 2021). Many drugs have been repurposed for the treatment of AD and PD with the advancements in understanding their targets and mechanisms of action which are highly required for drug approval and pharmacological applications (Iyaswamy et al., 2020b; Wang et al., 2021). Among these repurposed drugs, one TCM combination has recently shed light on the treatment of tauopathies in AD (Iyaswamy et al., 2020a). Yuan-Hu Zhi Tong (YZT), available on the market in capsule form, has been used for the treatment of psychiatric disorders and was recently repurposed for the treatment of tauopathies in AD as it can reduce the toxicities of insoluble phospho-Tau (pTau) *via* regulating ubiquitin proteasomal system (UPS) (Iyaswamy et al., 2020a). Among the various active phytochemicals, Protopine (PRO) is the only active small molecule which can enter the blood brain barrier (BBB) and activate proteasomal degradation of pathological tau *via* HDAC6 inhibition (Sreenivasmurthy et al., 2022b). Chemical engineering of PRO (PRO-Br) has enhanced its BBB permeability, bioavailability, and its precise mechanism of action *via* chaperone-mediated autophagy (CMA) (Sreenivasmurthy et al.). PRO-Br particularly reduced insoluble pathogenic pTau clumps and enhanced memory functions in AD mouse models (Sreenivasmurthy et al.). Protopine-derivative PRO-Br prompted CMA for the clearance of pTau and inhibited the function of HDAC6 to enhance the expression of molecular chaperones and lysosomal biogenesis.

**FIGURE 1:**
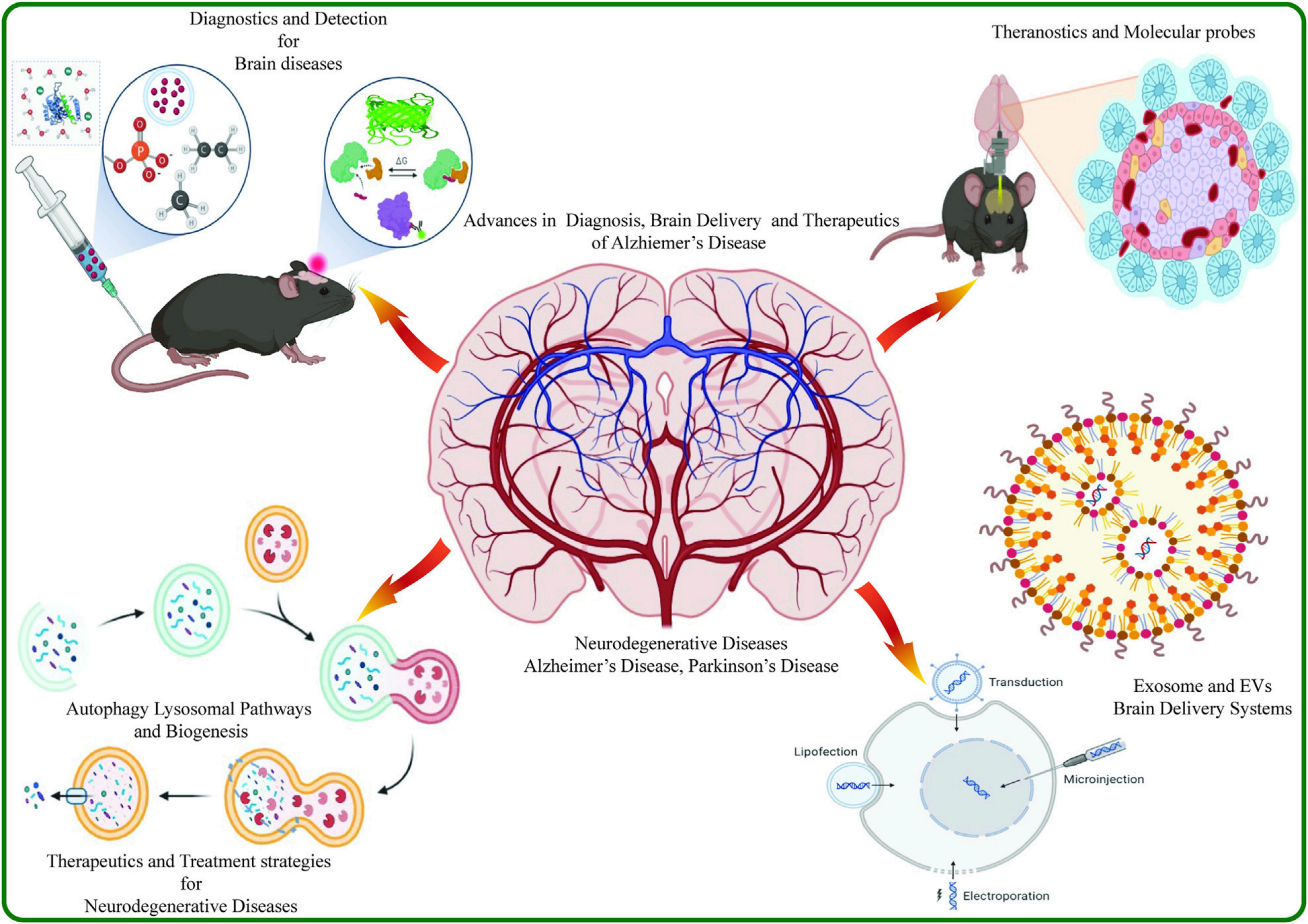


In another submitted article, the authors found that knockdown of kinesin I heavy chain KIF5B reduces tau hyperphosphorylation and aggregation in AD models with tauopathies (Selvarasu et al.). Overexpression of KIF5B significantly increased tau hyperphosphorylation, aggregation, and memory impairment in AD models suggesting that KIF5B is important for tau stability. Conclusively, their results found that KIF5B is an essential protein in modulating the tau firmness in microtubules and reducing the tau aggregates accumulation in AD and other tauopathies. One more research article evaluated the functions of differentially expressed genes (DEGs) and intestinal microbiota regarding the occurrence and causatives of post-stroke depression (PSD), which is one of the most common NDs (Li et al.). In this study, the authors identified metabolic pathways causing stroke and PSD *via* enrichment analysis, and constructed a global metabolic network to study the disease progression and the prevention of PSD. Based on transcriptomics, 16S rDNA sequencing and non-targeted metabolomics patient data, the authors found the causative microbial flora and impaired DEGs which can induce depression by impacting the metabolism of PSD patients. Collectively, using advanced technology, this study has found new therapeutic targets for the impediment of PSD and therapy for PSD patients with concluding evidence. An additional interesting study revealed that SIRT6 is an essential target for the neurological severity in Friedreich ataxia (FRDA), a recessive ND (Rodden et al.). Patients with FRDA were subject to DNA analysis to find the targets proteins which are contributory to the symptoms of FRDA and its severity. It was revealed that patients with cytosine variants of SIRT6 had less severe neurological symptoms and visual dysfunction when compared with the common thymine SIRT6 variant patients. In conclusion, the authors found that transcriptomic changes in the SIRT6 variant function may be causative of the neurological symptoms of FRDA patients, and a deep constructive analysis remains a requirement for clinical trials.

The research articles published in this Research Topic demonstrate the advancement in studies pertaining to NDs, utilizing state-of-the-art technologies and strategies with effective tools for molecular therapeutic research. These research articles may provide a foundation for future research ideas and may enable the development of therapeutic targets with translational potential in ND research. In conclusion, this Research Topic is noteworthy in its contribution to the advancement of diagnostics and therapeutics of NDs.
